# Prognosis of primary pulmonary adenocarcinoma after surgical resection in small‐breed dogs: 52 cases (2005‐2021)

**DOI:** 10.1111/jvim.16739

**Published:** 2023-05-25

**Authors:** Masanao Ichimata, Yumiko Kagawa, Keita Namiki, Atsushi Toshima, Yuko Nakano, Fukiko Matsuyama, Eri Fukazawa, Kei Harada, Ryuzo Katayama, Tetsuya Kobayashi

**Affiliations:** ^1^ Japan Small Animal Cancer Center, Public Interest Incorporated Foundation Japan Small Animal Medical Center Tokorozawa Saitama Japan; ^2^ North Lab Sapporo Hokkaido Japan; ^3^ Public Interest Incorporated Foundation Japan Small Animal Medical Center Tokorozawa Saitama Japan; ^4^ Veterinary Cancer Center, Hayashiya Animal Hospital, Uji Kyoto Japan

**Keywords:** adjuvant chemotherapy, pneumonectomy, pulmonary adenocarcinoma, small‐breed dogs

## Abstract

**Background:**

Tumor size is an important prognostic factor in lung cancer in dogs, and the canine lung carcinoma stage classification (CLCSC) recently has been proposed to subdivide tumor sizes. It is unclear if the same classification scheme can be used for small‐breed dogs.

**Objectives:**

To investigate whether the tumor size classification of CLCS is prognostic for survival and progression outcomes in small‐breed dogs with surgically resected pulmonary adenocarcinomas (PACs).

**Animals:**

Fifty‐two client‐owned small‐breed dogs with PAC.

**Methods:**

Single‐center retrospective cohort study conducted between 2005 and 2021. Medical records of dogs weighing <15 kg with surgically resected lung masses histologically diagnosed as PAC were examined.

**Results:**

The numbers of dogs with tumor size ≤3 cm, >3 cm to ≤5 cm, >5 cm to ≤7 cm, or >7 cm were 15, 18, 14, and 5, respectively. The median progression‐free interval (PFI) and overall survival time (OST) were 754 and 716 days, respectively. In univariable analysis, clinical signs, lymph node metastasis, margin, and histologic grade were associated with PFI, and age, clinical signs, margin, and lymph node metastasis were associated with OST. Tumor size classification of CLCS was associated with PFI in all categories, and tumor size >7 cm was associated with OST. In multivariable analysis, tumor size >5 cm to ≤7 cm and margin were associated with PFI, and age was associated with OST.

**Conclusions and Clinical Importance:**

The tumor size classification of CLCS would be an important prognostic factor in small‐breed dogs with surgically resected PACs.

**Abbreviations**
AEs: adverse events
CBDCA: carboplatin
CI: confidence interval
CLCSC: canine lung carcinoma stage classification
CT: computed tomography
HR: hazard ratio
IQR: interquartile range
JSACC: Japan Small Animal Cancer Center
MDCT: multidetector‐row computed tomography
OST: overall survival time
PAC: primary pulmonary adenocarcinoma
PD: progressive disease
PFI: progression‐free interval
PPC: primary pulmonary carcinoma
PPN: primary pulmonary neoplasia
TOC: toceranib phosphate
VCOG‐CTCAE: Veterinary Comparative Oncology Group Common Terminology Criteria for Adverse Events

## 1 INTRODUCTION

Primary lung cancer is a common malignancy in people^1^ but is relatively rare in dogs.^2^ Among lung tumors in dogs, primary pulmonary carcinomas (PPCs) are most common, and among PPC cases, pulmonary adenocarcinomas (PACs) are reported to account for 60%‐80%.^3^, ^4^, ^5^, ^6^

Prognostic factors for lung cancer in people include histologic classification,^7^, ^8^, ^9^ performance status,^9^, ^10^ age,^10^ sex,^10^, ^11^ smoking,^9^, ^12^ molecular biology signatures,^13^ and stage classification. The International Association for the Study of Lung Cancer recently has published a revised version of the tumor‐node‐metastasis stage groupings used for lung cancer in human patients.^14^ Tumor size was further subdivided in the revised version, indicating that it is an important prognostic factor in lung cancer patients.

Although a staging system for lung tumors in dogs was proposed by the World Health Organization in 1980, this staging system did not include an assessment of tumor size. However, tumor volume^15^, ^16^, ^17^ and longest tumor diameter^6^, ^17^ have been reported to be associated with survival in dogs with PPC, suggesting that tumor size might be an important prognostic factor in dogs. In 2020, the canine lung carcinoma stage classification (CLCSC) based on stage classification in humans was proposed.^18^ In the CLCSC, tumor size was subdivided into 4 categories: ≤3 cm, >3 cm to ≤5 cm, >5 cm to ≤7 cm, and >7 cm. Each CLCSC component, tumor size, lymph node metastasis, and distant metastasis was associated with survival time in PPC of dogs, suggesting that the CLCSC is an important prognostic factor for dogs with PPC. Subsequently, a relationship between CLCSC and survival also was observed in a larger population of dogs.^6^

However, unlike humans, dog breeds have large differences in body size. In the 2 reports that evaluated CLCSC, the median weight of the dogs was >20 kg.^6^, ^18^ To our knowledge, the prognosis of surgically resected PACs in small‐breed dogs has not been clarified, and whether the tumor size classification of CLCS could be applied to PAC of small‐breed dogs has not been examined. We hypothesized that the tumor size classification of CLCS would have a prognostic impact on PAC in dogs weighing <15 kg.

Therefore, we aimed to investigate whether the tumor size classification of CLCS is prognostic for survival and progression outcomes in small‐breed dogs with surgically resected PACs. We also explored other potential risk factors related to progression outcomes and survival times.

## 2 MATERIALS AND METHODS

### 2.1 Case selection

A retrospective cohort study was performed at the Japan Small Animal Cancer Center (JSACC) between April 1, 2005, and March 31, 2021, in dogs weighing <15 kg that had undergone surgical resection of pulmonary masses diagnosed as PACs by histologic examination. Dogs with a prior history of adenocarcinoma and concomitant malignancies other than PACs on the day of initial examination were excluded.

### 2.2 Medical records review

Information from medical records was obtained on signalment, clinical signs at initial examination, clinical pathology results, including hematology and urinalysis, radiography, abdominal ultrasonography, computed tomography (CT), cytology, histology, postoperative chemotherapy, date that tumor recurrence occurred, and presence of distant metastases. Staging, including history, physical examination, blood test results, urinalysis, thoracic and abdominal radiography, and abdominal ultrasound examination, was performed in all cases. The diagnostic methods and date and cause of death (PAC‐related, other causes, unknown causes, and last day of confirmed survival if the date of death was unknown) also were collected. When follow‐up data were limited, medical records were obtained from the primary veterinarian, or updates were obtained by telephone from the owner. The presence of clinical signs was defined as the initial presentation with any potential clinical sign associated with a lung tumor, including coughing, tachypnea, dyspnea, lethargy, exercise intolerance, anorexia, or hemoptysis, whereas no clinical signs at diagnosis were defined as incidental identification of a lung tumor on imaging with no clinical signs associated the lung tumor.^18^ The CLCSC system was used for staging and tumor size classification.^15^ The anatomic location and longest tumor diameter of PAC and lymph node status were recorded according to the CT data that was reassessed by a single diagnostic radiologist (A.T). If the CT data were incomplete, the data were supplemented with the original diagnostic imaging reports. If heterogeneous contrast‐enhanced lymph node patterns were seen, the longest lymph node diameter was >12 mm, or a lymph node/vertebral body ratio was >1.05 on CT, lymph node metastasis was suspected.^19^ Diagnosis, lymph node status, presence, and location of intrapulmonary nodules separate from the primary tumor, tumor invasion into specific organs, and surgical margins were recorded from the original histopathologic reports. Because the histologic pattern and grade of PAC were not evaluated in the original histopathology report, those were reassessed by 2 pathologists (Y.K and K.N). The PAC histologic grade was based on the PPC grading system.^20^ Complete resection was defined as a lack of tumor cells at the margins. Perioperative death was defined as death within 14 days after surgery. Adjuvant chemotherapy was defined as cytotoxic chemotherapy administered within 4 weeks after surgery. Surgical complications and adverse events (AEs) of adjuvant chemotherapy were assessed using the Veterinary Cooperative Oncology Group Common Terminology Criteria for Adverse Events (VCOG‐CTCAE) version 1.1.^21^

### 2.3 Computed tomography examination

For the CT scanner at the JSACC, we used a 4 multidetector‐row (MD) CT scanner (Asteion Super4 Edition TSX‐02B/6; Toshiba Medical Systems, Otahara City, Japan) from 2005 to January 2015 and an 80MDCT scanner (Aquilion prime TSX‐303A; Toshiba Medical Systems, Otahara City, Japan) from February 2015 to 2021. Contrast‐enhanced CT of the chest was performed under general anesthesia. The CT scanning and reconstruction parameters used at the JSACC for the 4MDCT scanner were 120 kVp, 120 mA, rotation time = 0.75 seconds, slice thickness = 1‐2 mm, beam pitch = 1.374, reconstruction interval = 1‐2 mm, and a standard algorithm, whereas for the 80MDCT scanner these were 120 kVp, 350 mA, rotation time = 0.5 seconds, slice thickness = 0.5 mm, beam pitch = 1.388, reconstruction interval = 0.5 mm, and a standard algorithm. Iopamidol (Oypalomin; Fuji Pharma) was used as the contrast agent for CT. This contrast agent was administered at a dosage of 2‐2.5 mL/kg (600‐750 mg/kg) for >10 seconds via the cephalic vein, and images were taken 120 seconds after administration.

### 2.4 Statistical analysis

Progression‐free interval (PFI) was defined as the time from surgery to progressive disease (PD). Progressive disease was defined as recurrence, dissemination, metastasis, or malignant pleural effusion noted postoperatively and was confirmed when the diagnosis was based on imaging, cytologic findings or histologic findings. Dogs were censored from PFI analysis if no detectable PD was noted at the last follow‐up or death. Overall survival time (OST) was defined as the time from surgery to death from any cause. Dogs that were lost to follow‐up or remained alive were censored from OST analysis at the date of the last contact.

The Kaplan‐Meier method was used to estimate and depict PFI and OST. Interval or survival between groups was compared using the log‐rank method. Age, weight, clinical signs at the initial examination, lymph node status (N0 vs N1, 2), completeness of tumor resection (margin), histologic grade (grade 1‐3), histologic patterns (papillary vs other), tumor size classification of CLSC (≤3 cm, >3 cm to ≤5 cm, >5 cm to ≤7, or >7 cm), and adjuvant chemotherapy were analyzed as variables to identify potential risk factors associated with PFI and OST. These data were reported as median and interquartile range (IQR). Lymph node metastasis was evaluated by histopathologic examination, and as in previous reports, for dogs in which lymph node resection or biopsy was not performed, lymph node status was recorded as N0.^6^, ^18^, ^20^ Dogs that died perioperatively were excluded from the analysis of PFI and OST of adjuvant chemotherapy. To determine potential risk factors associated with PFI and OST, univariable and multivariable analyses were performed using the Cox proportional hazards regression model. Multivariable analyses included variables for which *P* < .05 was obtained in univariable analyses. These results were presented as hazard ratio (HR) and 95% confidence intervals (CI). The potential risk factors between the dogs receiving or not receiving adjuvant CBDCA were compared using Fisher's exact test. *P* values <.05 were considered significant. Statistical analysis was performed using EZR software (Saitama Medical Centre, Jichi Medical University, Saitama, Japan).

## 3 RESULTS

### 3.1 Patient population

Fifty‐two client‐owned small‐breed dogs met the criteria for inclusion. There were 23 castrated males, 26 spayed females, and 3 intact males. The median age was 12 years (IQR, 10.0‐13.0 years). Seventeen dog breeds were included: Toy Poodle (n = 10), Miniature Dachshund (n = 10), Chihuahua (n = 7), Maltese (n = 4), Shih Tzu (n = 4), and 17 dogs of other breeds, each with fewer than 3 dogs per breed (n = 17). The median body weight was 5.6 kg (IQR, 4.4‐7.0 kg) and the numbers of dogs ≤5 kg, >5 kg to ≤10 kg, and >10 kg to ≤15 kg were 19, 29, and 4, respectively. Clinical signs at initial presentation were noted in 33 dogs (63.5%): coughing (n = 32), anorexia (n = 9), exercise intolerance (n = 9), lethargy (n = 3), hemoptysis (n = 2), tachypnea (n = 1), and dyspnea (n = 1).

### 3.2 Diagnostic testing and staging

Contrast‐enhanced thoracic CT was performed in all cases. Three patients had CTs performed at other institutions. The CT data were partially or totally lost for 2 dogs and could not be reviewed, and thus the description from the original diagnostic imaging report was used. Those reports described the anatomic location and the longest diameter of the mass but did not describe the details of the lymph nodes. The median longest diameter of the lung masses was 41.0 mm (IQR, 30.0‐54.0 mm). The lung mass was solitary in 49 dogs, and satellite nodules were found in the same lobes as the lung mass in 3 dogs. The numbers of dogs with tumor size ≤3 cm, >3 cm to ≤5 cm, >5 cm to ≤7 cm, or >7 cm were 15 (28.8%), 18 (34.6%), 14 (26.9%), and 5 (9.6%), respectively. Lung masses occurred in the cranial lobe of the right lung in 8 dogs (15.4%), the middle lobe of the right lung in 5 dogs (9.6%), the caudal lobe of the right lung in 13 dogs (25.0%), the accessory lobe in 3 dogs (5.8%), the cranial lobe of the left lung in 15 dogs (28.8%), and the caudal lobe of the left lung in 8 dogs (15.4%). Fourteen of 50 dogs (28.0%) were diagnosed with suspected lymph node metastasis. Other CT findings included a small amount of effusion in 4 dogs, 1 of which had lung lobe torsion. Pleural fluid examinations were performed in 2 of 4 dogs; both dogs were found to have transudative pleural effusion and no tumor cells. Distant metastases were not found in any of the cases.

### 3.3 Surgery

Forty‐six dogs underwent resection of 1 lung lobe. Because of the anatomical location of the tumor and the pulmonary vessels and trachea, 3 dogs underwent resection of the accessory lobe and right caudal lung lobe, and 3 dogs underwent left lung resections. Lymph nodes were removed in 37 dogs (71.2%); the regional tracheobronchial lymph nodes were removed in 35 dogs. In the other 2 dogs, cranial mediastinal and sternal lymph nodes were removed in 1 dog, and cranial mediastinal lymph nodes were removed in the other dog. One dog had a nodule in the caudal mediastinum that was removed. Lymph nodes were removed in 12 of 14 dogs with suspected metastases on CT. Intraoperative complications occurred in 6 (11.5%) dogs, postoperative complications occurred in 19 (36.5%) dogs, and perioperative death was observed in 4 dogs (7.7%; Table 1).

**TABLE 1:** Intra‐ and postoperative complications in small‐breed dogs with surgically resected pulmonary adenocarcinomas.

Complications	Grade	No. of dogs
Intraoperative		
Minor hemorrhage	1	4
Air leakage	1	1
Hemorrhage	2	1
Postoperative		
Seroma	1	4
Subcutaneous emphysema	1	5
Reaccumulation of pleural effusion after removal of chest drain	1	2
Hypoxemia requiring oxygen supplementation (>24 h)	3	1
Acute congestive heart failure (pulmonary edema)	3	1
Aspiration pneumonia	3	1
Pleural effusion requiring repeated thoracocentesis	4	1
Hemorrhage requiring revision surgery	4	1
Suspected acute congestive heart failure	5	1
Unknown cause of death	5	1
Acute respiratory distress syndrome and disseminated intravascular coagulation	5	1
Acute respiratory distress syndrome because of noncardiogenic pulmonary edema	5	1

Postoperatively, clinical signs improved in 27 of 33 dogs (84.3%). Six dogs showed no improvement in coughing after surgery, and the suspected causes of coughing (including causes affecting >1 dog) were tracheal or bronchial‐related disease (n = 3), myxomatous degenerative mitral valve disease (n = 3), carcinomatous pleuritis (n = 1), pneumonia (n = 1), and pneumothorax (n = 1). The remaining 3 dogs, in which improved coughing could not be confirmed, died during the perioperative period, and therefore it was not possible to assess whether coughing had improved.

### 3.4 Histology and CLCSC staging

The primary PAC was completely resected in 45 dogs (86.5%). The histologic grades of PAC were grade 1, 2, and 3 in 35 (67.3%), 15 (28.9%), and 2 (4%) dogs, respectively. Vascular invasion was noted in 8 (15.4%) dogs. There were 14, 17, 13, and 8 dogs for the T1, T2, T3, and T4 stages of CLCSC, respectively. In 5 dogs, T stage did not correlate with the tumor size classification of CLSC because of the presence of either intrathoracic organ invasion or a lung nodule. Lymph node metastases were observed in 9 of 37 dogs (24.3%), of which 6 dogs had suspected lymph node metastases on CT. Thirteen, 28, and 11 dogs were classified as CLCSC stages 1, 2, and 3, respectively. No dogs were classified as stage 4.

### 3.5 Chemotherapy

Adjuvant chemotherapy was administered to 19 dogs, and carboplatin (CBDCA) was used in all dogs. The CBDCA was administered to 3 of the 19 dogs by a referring veterinarian. At the JSACC, CBDCA was administered IV 4‐6 times at an interval of every 3 weeks and at a dosage of 200‐250 mg/m^2^ for 30‐60 minutes. The CBDCA administration had been proposed to the owner at the discretion of each attending or referring veterinarian. A median initial dose of 225 mg/m^2^ (range, 180‐250 mg/m^2^) was administered, and dogs received a median of 4 doses (range, 1‐6). Grade 4 neutropenia and thrombocytopenia occurred in 10 (58.8%) and 8 (42.1%) dogs, respectively. Neutropenia with fever occurred in 6 dogs (31.6%), with all dogs recovering. Grade 3 or 4 gastrointestinal toxicity occurred in 2 dogs (10.5%). Differences in baseline prognostic factors were present between dogs that did and did not receive chemotherapy (Table 2).

**TABLE 2:** Comparisons of potential risk factors in small‐breed dogs with pulmonary adenocarcinomas treated with surgery alone or surgery plus adjuvant chemotherapy.

Variables	Number	Surgery only number (%)	Surgery + chemotherapy number (%)	*P*‐value
Age, years Median	48	12.0	10.0	.05
Body weight, kg Median	48	5.3	6.3	.05
Clinical signs				
No	18	10 (55.6%)	8 (44.4%)	.76
Yes	30	19 (63.3%)	11 (36.7%)	
Tumor size classification				
≤3 cm	13	11 (84.6%)	2 (15.4%)	.16
>3 cm to ≤5 cm	16	9 (56.3%)	7 (73.7%)	
>5 cm to ≤7 cm	14	6 (42.9%)	8 (57.1%)	
>7 cm	5	3 (60.0%)	2 (40.0%)	
N stage				
N0	40	23 (57.5%)	17 (42.5%)	.45
N1 or 2	8	6 (75.0%)	2 (25.0%)	
Margin				
Complete	42	26 (61.9%)	16 (38.1%)	.67
Incomplete excision	6	3 (50.0%)	3 (50.0%)	
Histologic classification				
Papillary	41	26 (63.4%)	15 (36.6%)	.41
Others	7	3 (42.9%)	4 (57.1%)	
CLCSC stage				
Stage 1	11	10 (%)	1 (%)	.07
Stage 2	26	13 (%)	13 (%)	
Stage 3	11	6 (%)	5 (%)	

Abbreviation: CLCSC, canine lung carcinoma stage classification.

One dog did not receive adjuvant CBDCA but was given thalidomide postoperatively. The affected dog had multiple lung metastases on Day 193 and died on Day 220.

### 3.6 Outcomes

Regarding outcomes at the time of analysis, 41 dogs had died (78.8%), 6 were still alive (11.5%), and 5 were lost to follow‐up (9.6%).

Progressive disease of PAC was reported postoperatively in 24 dogs (46.2%). The sites of PD after surgery were as follows (including sites affecting multiple dogs): lung (n = 13), malignant pleural effusion (n = 6), pleura (n = 3), humerus (n = 2), ribs (n = 2), dorsal mediastinum (n = 1), subcutaneous (n = 1) and right eye (n = 1). Progressive disease was diagnosed by diagnostic imaging alone in 8 dogs, and in the remaining 16 dogs, PD was diagnosed by diagnostic imaging and cytology in 14 dogs and diagnostic imaging and histopathology in 2 dogs. The median time from surgery to PD was 208 days (IQR, 52‐387.3 days). Twenty‐two of the dogs with PD had died by the date of the last confirmation. The causes of death in these dogs were PAC (n = 18), non‐neoplastic diseases (n = 2), and other neoplastic diseases (oral malignant melanoma, n = 1), and in 1 case, the cause of death was unknown. Non‐neoplastic diseases were congestive heart failure (n = 1) and sepsis (n = 1) associated with pyelonephritis.

Except for dogs that died in the perioperative period, 16 of 25 dogs without PD had died. The causes of death in these dogs were suspected to be non‐neoplastic disease (n = 9) and other neoplastic diseases (cutaneous epitheliotropic lymphoma, n = 1), and the cause of death in 6 dogs was unknown. Non‐neoplastic diseases included congestive heart failure (n = 2), seizures (n = 2), immune‐mediated hemolytic anemia (n = 1), obstructive hydrocephalus (n = 1), pneumonia (n = 1), chronic kidney disease (n = 1), and protein‐losing enteropathy (n = 1).

**FIGURE 1:**
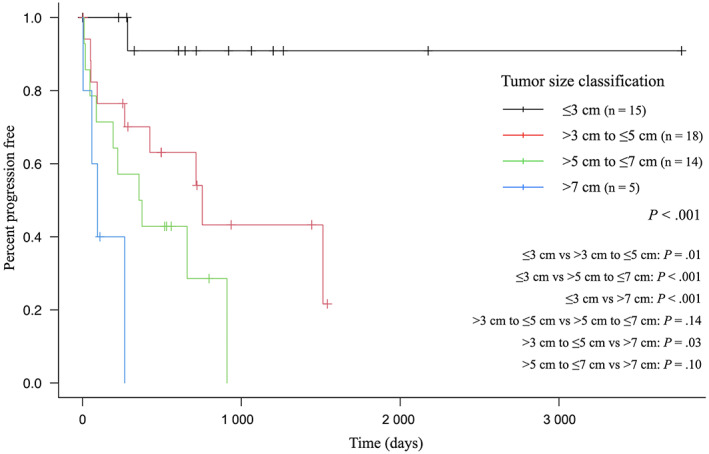
Kaplan–Meier curve showing the progression‐free interval of small‐breed dogs with surgically resected pulmonary adenocarcinomas by tumor size classification. Significant differences were found between ≤3 cm and >3 cm to ≤5 cm (*P* = .01), ≤3 cm and >5 cm to ≤7 cm (*P* < .001), ≤3 cm and >7 cm (*P* < .001), and >3 cm to ≤5 cm and >7 cm (*P* = .03). Vertical lines indicate censoring.

**FIGURE 2:**
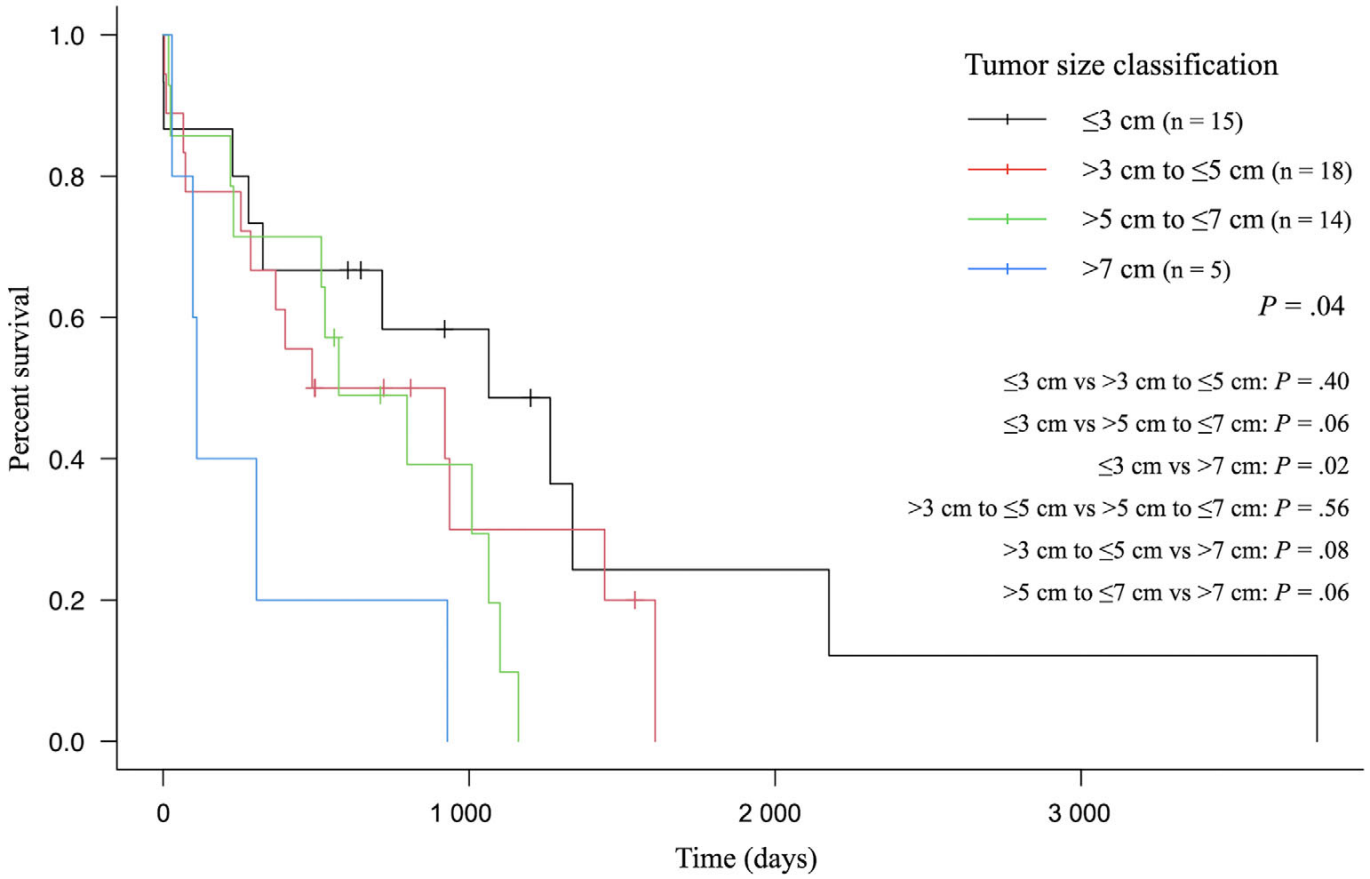
Kaplan–Meier curve showing the overall survival time of small‐breed dogs with surgically resected pulmonary adenocarcinomas by tumor size classification. A significant difference was found only between ≤3 cm and >7 cm (*P* = .02). Vertical lines indicate censoring.

Median PFI and OST were 754 (95% CI, 354‐Not reached days) and 716 days (95% CI, 326‐1009 days), respectively. Median PFIs for dogs with tumor sizes ≤3 cm, >3 cm to ≤5 cm, >5 cm to ≤7 cm, or >7 cm were not reached, 754, 365.5, and 95 days, respectively (Figure 1). Median OSTs for dogs with tumor sizes ≤3 cm, >3 cm to ≤5 cm, >5 cm to ≤7 cm, or >7 cm were 1064, 704, 574, and 110 days, respectively (Figure 2). The 1‐year survival rate was 63.5%, and the 2‐year survival rate was 49.0%. Median follow‐up time was 544 days (IQR, 229.3‐954 days). Median follow‐up time for censored dogs was 710 days (IQR, 581.5‐865 days).

Results of univariable and multivariable analyses investigating the association of PFI and OST with potential risk factors are shown in Tables 3 and 4, respectively. In univariable analysis, potential risk factors associated with PFI were clinical signs, lymph node metastasis, margin, and histologic grade, and those associated with OST were age, lymph node metastasis, and margin. Tumor size classification of CLCS was associated with PFI in all categories, and only tumor size >7 cm was associated with OST. In multivariable analysis, potential risk factors associated with PFI were tumor size >5 cm to ≤7 cm (adjusted HR, 13.68; 95% CI, 1.66‐112.5; *P* = .01) and margin (adjusted HR, 5.85; 95% CI, 1.45‐23.51; *P* = .01), and the only potential risk factor associated with OST was age (adjusted HR, 1.28; 95% CI, 1.07‐1.53; *P* = .01).

**TABLE 3:** Univariable Cox regression analysis of potential risk factors for the progression‐free interval and overall survival time in 52 small‐breed dogs with surgically resected pulmonary adenocarcinomas.

Variable	N	Progression‐free interval		Overall survival time	
		Hazard ratio (95% CI)	*P*‐value	Hazard ratio (95% CI)	*P*‐value
Age	52	1.07 (0.88‐1.31)	.48	1.27 (1.07‐1.50)	.01
Body weight	52	0.97 (0.83‐1.13)	.69	0.92 (0.80‐1.06)	.26
Clinical signs					
No	19	Reference		Reference	
Yes	33	2.67 (1.05‐6.81)	.03	2.13 (1.05‐4.29)	.03
Tumor size classification	52		<.001		.04
≤3 cm	15	Reference		Reference	
>3 cm to ≤5 cm	18	8.87 (1.12‐70.09)	.04	1.62 (0.68‐3.81)	.27
>5 cm to ≤7 cm	14	17.49 (2.19‐139.80)	.007	2.13 (0.87‐5.23)	.10
>7 cm	5	43.12 (4.47‐416.20)	.001	4.76 (1.51‐15.06)	.01
Lymph node status					
N0	43	Reference		Reference	
N1, 2	9	3.44 (1.33‐8.90)	.02	2.2 (1.03‐4.70)	.04
Margin					
Complete	45	Reference		Reference	
Incomplete	7	12.54 (4.37‐36.02)	<.001	2.92 (1.25‐6.82)	.01
Histologic grade	52		.002		.20
Grade 1	35	Reference		Reference	
Grade 2	15	3.09 (1.31‐7.27)	.01	1.78 (0.90‐3.52)	.10
Grade 3	2	7.36 (1.57‐34.56)	.01	2.40 (0.56‐10.41)	.24
Histologic classification					
Papillary	45	Reference		Reference	
Others	7	0.56 (0.16‐1.92)	.36	0.44 (0.18‐1.11)	.08
Adjuvant chemotherapy					
No	19	Reference		Reference	
Yes	29	1.33 (0.58‐3.05)	.50	0.78 (0.39‐1.53)	.46

Abbreviation: CI, confidence interval.

**TABLE 4:** Multivariable Cox regression analysis of potential risk factors for the progression‐free interval and overall survival time in 52 small‐breed dogs with surgically resected pulmonary adenocarcinomas.

Variable	N	Progression‐free interval		Overall survival time	
		Hazard ratio (95% CI)	*P*‐value	Hazard ratio (95% CI)	*P*‐value
Age	52	‐	‐	1.28 (1.07‐1.53)	.01
Clinical signs					
No	19	Reference		Reference	
Yes	33	1.42 (0.45‐4.51)	.55	1.82 (0.80‐4.14)	.15
Tumor size classification					
≤3 cm	15	Reference		Reference	
>3 cm to ≤5 cm	18	5.74 (0.69‐47.52)	.11	1.67 (0.69‐4.04)	.25
>5 cm to ≤7 cm	14	13.68 (1.66‐112.50)	.01	1.51 (0.57‐4.00)	.41
>7 cm	5	6.55 (0.47‐91.08)	.16	2.00 (0.47‐8.42)	.35
Lymph node status					
N0	43	Reference		Reference	
N1, 2	9	2.40 (0.75‐7.66)	.14	2.31 (0.99‐5.41)	.05
Margin					
Complete	45	Reference		Reference	
Incomplete	7	5.85 (1.45–23.51)	.01	1.20 (0.40‐3.55)	.75
Histologic grade					
Grade 1	35	Reference		‐	
Grade 2	15	2.61 (0.94‐7.23)	.07	‐	‐
Grade 3	2	1.94 (0.20‐18.99)	.57	‐	‐

Abbreviation: CI, confidence interval.

### 3.7 Outcome after disease progression

Of the 24 dogs with PD, 12 dogs received rescue treatment: toceranib phosphate (TOC; n = 9), surgery (n = 2), and CBDCA (n = 1). Surgeries on 2 dogs were performed by referring veterinarians; 1 dog underwent right eye removal, and the other underwent lobectomy of the right caudal lung lobe, both of which were diagnosed as PAC metastases on histopathologic examination. One dog that had received CBDCA as the primary rescue treatment had TOC as a further rescue treatment after recurrence of malignant pleural effusion.

## 4 DISCUSSION

In our study, Median PFI and OST for surgically resected PACs in small‐breed dogs were 754 and 716 days, respectively. In multivariable analysis, tumor size >5 cm to ≤7 cm and margin were associated with PFI, and age was associated with OST. Risk factors associated with progression outcomes and survival time in small‐breed dogs with surgically resected PACs have not been reported previously.

In small‐breed dogs with surgically resected PACs, tumor size classification of CLCS was associated with PFI. No significant difference was found between tumor sizes >3 cm to ≤5 cm and >5 cm to ≤7 cm, and between >5 cm to ≤7 cm and >7 cm. However, these results potentially could be attributed to Type II statistical error resulting from inadequate sample size. In addition, because the inclusion criteria in our study were limited to dogs in which surgical resection was performed, non‐surgical treatment might have been selected for dogs with larger tumor sizes. Overall survival time was not associated with tumor size classification of CLCS. In our study, the association between tumor size classification of CLCS and OST may have been influenced by the fact that 16 of 48 dogs (33.3%), excluding deaths in the perioperative period, died without PD of PACs and half of the PD cases received rescue treatment.

The median OST for our study was 716 days, indicating relatively long survival. The median survival time for surgically resected PPCs in dogs has been reported to be 92‐399 days^6^, ^18^, ^20^, ^22^, ^23^ and 131‐339 days for PACs.^17^, ^24^, ^25^ Although it is difficult to identify the specific reasons for long‐term survival, population characteristics defined by inclusion criteria (ie, restricted to PACs and small‐breed dogs) and the absence of cases with distant metastases were considered factors for long‐term survival in our study. Additionally, age was associated with OST in both univariable and multivariable analyses, with a 1.28‐fold increased risk of death for each additional year. In general, the number of dogs with comorbidities increases with age. The median age at initial presentation in our study was 12 years, and the dogs survived for a relatively long time after surgery, which suggests that the risk of death from diseases other than PAC increases with age and that age may have been associated with OST.

Surgical margins were associated with PFI in both univariable and multivariable analyses in our study. Previous studies have reported that surgical margins of PPCs or primary pulmonary neoplasia (PPN) are associated with OST^6^, ^17^, ^18^ and tumor‐specific survival^17^, ^18^ in univariable analysis but not in multivariable analysis.^6^, ^18^ These reports suggested that increased tumor size and invasiveness and location within the lung lobes (hilar and peripheral) may have been associated with surgical margins and influenced the results of multivariable analysis.^6^, ^18^ We only investigated some of those previously reported factors, making it difficult to determine the confounding factors between surgical margins and the other factors used in our study. All 7 dogs in our study with incomplete margins were found to have PD postoperatively, and the PFI was significantly shortened. Therefore, when surgical margins are incomplete, postoperative progression should be carefully monitored, and adjuvant therapy should be considered.

The lymph node metastatic rate determined by histology in our study was 24.3%, which was comparable to previously reported lymph node metastatic rates ranging from 22.4% to 32.7% for PPCs.^16^, ^18^, ^24^ Previous reports found that N stage affects survival in PPCs or PPNs.^16^, ^18^, ^20^, ^24^, ^25^ In our study, N stage was associated with PFI and OST in univariable analysis but not in multivariable analysis. However, in our study, 15 dogs in which lymph nodes could not be resected were considered N0. Therefore, histologic lymph node evaluation was not available in all dogs, and the impact of lymph node metastasis may have been underestimated. Interestingly, in 3 of the 9 dogs with histologic lymph node metastasis, no abnormalities were found in the lymph nodes on CT or observed intraoperatively. Similarly, the previous study of PPN reported discrepancies between preoperative CT and histology finding in 28.1% of dogs.^6^ Therefore, in the future, to accurately determine the prognostic impact of lymph node metastasis of PAC in small‐breed dogs, the regional lymph nodes should be removed regardless of preoperative CT and intraoperative macroscopic findings.

In small‐breed dogs with surgically resected PACs, we found PD in 46.2% of the dogs. The incidence of PD in dogs with PPC has been reported previously to be 43.3%‐50.7%,^18^, ^20^ which was comparable to what we observed. Therefore, adjuvant chemotherapy should be considered, especially in dogs with poor prognostic factors. To date, the benefit of adjuvant chemotherapy in dogs with PPC has not been shown.^6^, ^16^, ^18^ In our study, CBDCA was used in all dogs that received adjuvant chemotherapy, and no significant differences in PFI and OST were observed between the groups treated with surgery and CBDCA vs surgery alone. The retrospective nature of our study and lack of an analysis to avoid type II statistical error could be reasons for the lack of differences detected in our study. At our institution, we propose using adjuvant CBDCA in dogs with potential risk factors, such as advanced stage, tumor size >5 cm, and highly malignant PAC as determined by histology. In addition, we found significant differences for age and weight in dogs that received adjuvant CBDCA compared to those that did not. These selection biases could have influenced the observed effect of adjuvant CBDCA. Prospective studies should be conducted in larger populations to evaluate the effect of adjuvant CBDCA on PAC in dogs with potential risk factors.

Our study had some limitations. First, the study was conducted at a single institution with a relatively small sample size. Second, because of the retrospective study design, some diagnostic methods were incomplete; The CT equipment was modified during the study, and some data was lost; thus, there was incomplete reassessment of CT images. The distance between the surgical margins and the tumor on histologic tissue sections was not evaluated, and therefore the prognostic impact could not be assessed. Third, the PD evaluations were problematic; 9 dogs were diagnosed with PD using only thoracic radiography to identify lung masses. In these dogs, no cytologic or histologic assessments of the lung masses were performed. We also did not perform necropsies on these dogs. It is possible that the lung masses diagnosed as PD were diseases other than PAC. Routine monitoring was recommended, but the follow‐up schedule was not standardized. Fourth, because of the long study period, treatment methods were not consistent; surgical techniques for lobectomy were changed (eg, from suture ligation to Endo GIA Stapler) and rescue therapy options after PD were added (eg, TOC).

In conclusion, the tumor size classification of CLCS can be an important prognostic factor in small‐breed dogs with surgically resected PACs. Given that postoperative PD was found in 46.2% of the dogs in our study, a randomized trial with a larger sample size is warranted to determine the benefit of adjuvant chemotherapy for dogs with potential risk factors.

## CONFLICT OF INTEREST DECLARATION

Authors declare no conflict of interest.

## OFF‐LABEL ANTIMICROBIAL DECLARATION

Authors declare no off‐label use of antimicrobials.

## INSTITUTIONAL ANIMAL CARE AND USE COMMITTEE (IACUC) OR OTHER APPROVAL DECLARATION

Authors declare no IACUC or other approval was needed.

## HUMAN ETHICS APPROVAL DECLARATION

Authors declare human ethics approval was not needed for this study.
